# Correlation of age, sex and season with the state of human decomposition as quantified by postmortem computed tomography

**DOI:** 10.1007/s12024-021-00356-2

**Published:** 2021-02-15

**Authors:** Dominic L. C. Guebelin, Akos Dobay, Lars Ebert, Eva Betschart, Michael J. Thali, Sabine Franckenberg

**Affiliations:** 1 Institute of Forensic Medicine, University of Zurich, Zurich, Switzerland; 2 Institute of Diagnostic and Interventional Radiology, University Hospital Zurich, Zurich, Switzerland

**Keywords:** Virtopsy, Decomposition, Sociodemographic, Postmortem computed tomography, Radiological alteration index, Postmortem interval

## Abstract

Dead bodies exhibit a variable range of changes with advancing decomposition. To quantify intracorporeal gas, the radiological alteration index (RAI) has been implemented in the assessment of postmortem whole-body computed tomography. We used this RAI as a proxy for the state of decomposition. This study aimed to (I) investigate the correlation between the state of decomposition and the season in which the body was discovered; and (II) evaluate the correlations between sociodemographic factors (age, sex) and the state of decomposition, by using the RAI as a proxy for the extent of decomposition. In a retrospective study, we analyzed demographic data from all autopsy reports from the Institute of Forensic Medicine of Zurich between January 2017 to July 2019 and evaluated the radiological alteration index from postmortem whole-body computed tomography for each case. The bodies of older males showed the highest RAI. Seasonal effects had no significant influence on the RAI in our urban study population with bodies mostly being discovered indoors. Autopsy reports contain valuable data that allow interpretation for reasons beyond forensic purposes, such as sociopolitical observations.

## Introduction

The state of decomposition of a body varies considerably due to the many variables that impact the process of decay. These variations depend on environmental conditions such as temperature. Possible postmortem alterations consist of the body turning a greenish color, marbling of the skin, maggot infestation, bloating, mummification and skeletonization in the later stages [1]. Due to the large variety of postmortem changes and influencing factors, estimations of time of death are generally relatively vague and contain great variability [2]. Methods to determine the early postmortem interval (PMI) more precisely, such as thanato-chemical measurements of human cardiac troponin [3], are rarely used in practice and are mostly of academic interest [4]. Unfortunately methods to estimate the late PMI more accurately that apply a decomposition scoring system and integrate entomological data [5] suffer from shortcomings in statistical data analysis [6]. Ultimately, the complex estimation of the late postmortem interval usually still predominantly relies on the experience of the forensic pathologist [7].

In the last 20 years, postmortem computer tomography (PMCT) and postmortem magnetic resonance (PMMR) have become valuable adjuncts and even alternatives to conventional autopsy [8–10]. PMCT is sometimes considered superior to autopsy due to its ability to detect gas inside of a body [8, 11, 12]. Gas is a common radiological finding in decedents; it is either caused by vital embolism, i.e. after open or iatrogenic trauma, or develops postmortem due to decomposition [13]. For the latter, recently a method to quantify intracorporeal gas was established, named the radiological alteration index (RAI) for PMCT [14]. The RAI is a straightforward, visual grading system that considers seven different sites in the body with different weightings, allowing for the quantification of intracorporal gas and aiding in the distinction between postmortem decomposition, versus vital air embolism [14].

A subjective perception among the forensic pathologists at the Swiss Institutes of Forensic Medicine is that there tends to be an increase in cases of advanced decomposition during summer. This is thought to be caused by both a reduced discoverability of the diseased by the relatives due to the long summer holidays (they last six weeks in Switzerland and are the longest holidays throughout the year) and generally increased temperatures. In the hot summer months, bodies decay faster due to overall high temperatures, and show a more rapid infestation with maggots. Both are well-known factors that contribute to body decomposition [15–17].

It was furthermore shown that people with an insufficient social network, or who live alone, have an increased mortality risk if medical symptoms develop, as they lack a trusted person that would encourage medical attention [18]. Additionally, loneliness may be typical in older people with major health and mobility problems [19], which could lead to unrecognized death. In a forensic sample, this should be indicated in the autopsy data as an increased PMI and a state of advanced decomposition at detection.

The recently developed RAI allows for quantification of intracorporeal gas with a clearly defined value. Our aim was to evaluate two hypotheses by using the RAI as a proxy for the state of decomposition.

First, we examined whether there were seasonal influences on all autopsy case numbers (with decomposition gas) and the RAI.

Second, we examined whether there was a correlation between age or sex and the RAI.

## Material and methods

### Study design

For this retrospective study, all autopsy cases from January 1, 2017 to July 31, 2019 were retrieved from the Institute of Forensic Medicine of Zurich, resulting in 1178 cases. All cases underwent PMCT immediately after admission to our institute as standard routine procedure. After applying the exclusion criteria (open trauma and/or polytrauma), 440 cases (271 males, 169 females) remained. Demographic data, manner of death, and time of discovery of the body (season) were obtained directly from the autopsy report (overview in Table 1). The time difference between external examination on site and PMCT showed a mean of 0.77 days (standard deviation 0.79). Seasons were defined according to the astronomical definition (winter: December 21 to March 19; spring: March 20 to June 20; summer: June 21 to September 22 (in 2017 September 21); autumn: September 23 (in 2017 September 22) to December 20). The manner of death was specified as either natural, suicide, drug-related (suicide vs. accident) or non-natural due to other causes (homicide, accident, medical malpractice). All cases were also divided into two age groups (< 50 years, ≥ 50 years).

**Table 1:** Overview of the distribution of manner of death, subdivided into sex:

	Females	Males	Total
Natural	90	148	238
Suicide	16	15	31
Drug-related	24	53	77
Non-natural of other causes	39	55	94
Overall (RAI > 1)	169	271	440

### Postmortem computed tomography

PMCT was performed with a 128-slice scanner (SOMATOM Definition Flash, Siemens Healthineers, Erlangen, Germany) with bodies in the supine position using automatic dose modulation (CARE Dose 4D™, Siemens Healthineers, Erlangen, Germany). The imaging parameters were tube voltage, 120 kVp and slice collimation, 128 × 0.6 mm. PMCT images of the head and neck were reconstructed with a slice thickness of 1.0 mm and increment of 0.6 mm [20]. PMCT data review was performed on Syngo by imaging software for multimodality reading (Syngo. Via, Siemens Healthineers, Erlangen, Germany).

**Fig. 1:**
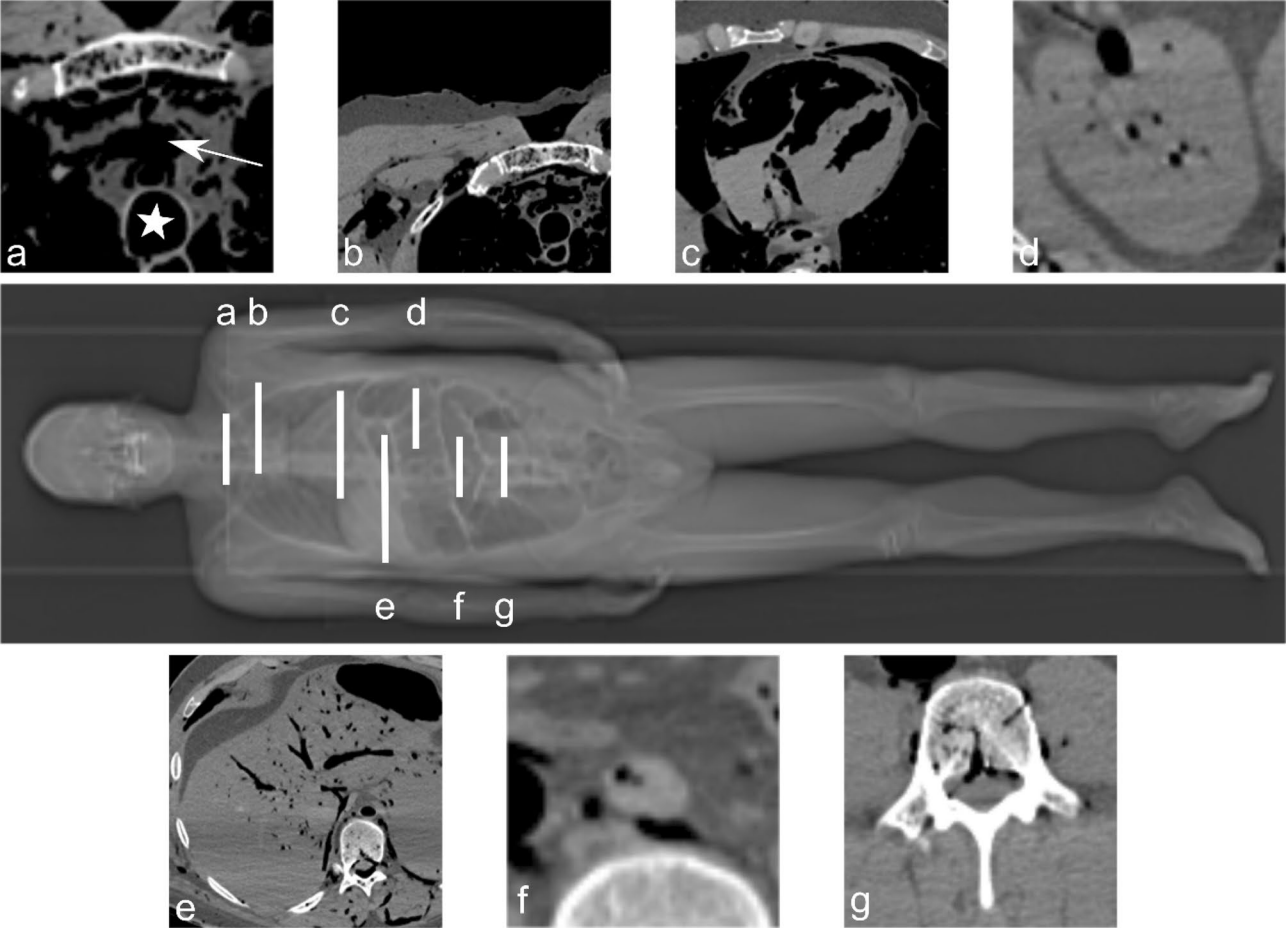
Calculation of the RAI for a male example case, with a total RAI score of 63. Grouped around the CT body scout view in the middle (white lines represent the position of the seven axial slices evaluated for the RAI, for better localization in slice a the innominate vein was marked with an arrow and the trachea with a star) are the magnified axial views of the **a** left innominate vein (grade 3, score 15), **b** subcutaneous pectoral tissue (grade 3, score 8), **c** heart cavities (grade 3, score 17), **d** kidney parenchyma (grade 1, score 0), **e** liver parenchyma and vessels (grade 2, score 5), **f** abdominal aorta (grade 1, score 8), and **g** vertebra L3 (grade 2, score 10)

To quantify the state of decomposition, we assumed a correlation of the state of decomposition with the volume of intracorporeal gas. To asses this intracorporeal gas, the RAI for each PMCT was retrospectively evaluated, as described by Egger et al. [14], by two medical students under the supervision of a board-certified forensic pathologist with 8 years of radiological experience. For each of the seven defined sites (as shown in Fig. 1) an initial visual grade of zero to three was assigned; each organ was then individually scored to determine the overall RAI (maximum 100). With the focus of this study being on decomposition states, cases with an RAI = 0 were excluded. A total of 284 out of 440 cases with an RAI > 0 remained.

### Statistics

The data did not have a normal distribution, and the groups were different sizes, therefore, Kruskal–Wallis H tests were performed to evaluate the correlations between age and/or season with the state of decomposition (as quantified by the RAI according to PMCT).

Linear regression was calculated for the RAI against age for both males and females respectively. To improve the comparison, two age groups were defined: a younger age group (< 50 years) and an older age group (≥ 50 years). Linear regression was calculated for the RAI against age for each sex.

Kruskal–Wallis tests were also performed to study the relation between sex and RAI, season and the overall number of cases. In addition, it was assessed if there are differences in the RAI of all younger age cases vs. the RAI of all older age cases, age of all male cases with an RAI < 50 vs. the age of all male cases with an RAI ≥ 50, and age of all female cases with an RAI < 50 vs. the age of all female cases with an RAI ≥ 50.

## Results

**Fig. 2:**
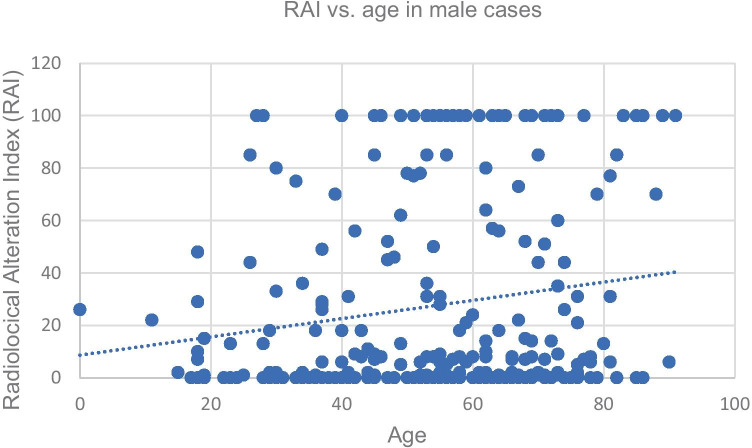
Radiological alteration index (RAI) vs. age for all male cases, slope: 0.35. Standard deviation of the residuals: Sy.x = 36.82 (y = 0.35x + 8.67)

**Fig. 3:**
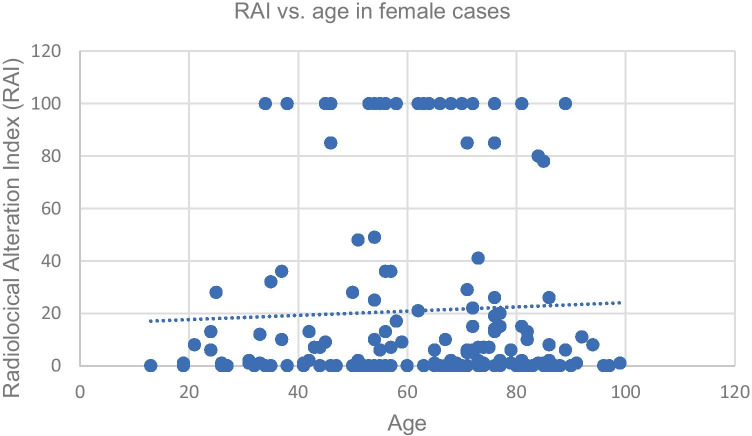
Radiological alteration index (RAI) vs. age for all female cases, slope: 0.1. Standard deviation of the residuals: Sy.x = 34.73 (y = 0.09x + 14.97)

The distribution of cases with an RAI > 0 per season can be seen in Table 2 (spring: 105, summer: 55, autumn: 41, winter: 81). The Kruskal–Wallis test results were not significant for all case numbers (RAI > 1) and seasons (Table 2); there was no significantly increased case number in any of the seasons. The Kruskal–Wallis test result was not significant for sex and RAI (Table 3); there was no correlation between the RAI and sex. A trend indicated that the younger age group was found with a lower RAI, suggesting a less decomposed state (RAI 31.58, error of the mean 3.71) than the older age group (RAI 41.49, error of the mean 2.89), but the Kruskal–Wallis test result was not significant (Table 3). There was a significant difference in age when comparing cases with an RAI ≥ 50 and an RAI < 50 (Table 3); bodies with an RAI ≥ 50 had a mean age of 60.86 yrs (error of the mean 1.56), while those with an RAI < 50 were slightly younger, with a mean age of 55.39 yrs (error of the mean 1.06). The Kruskal–Wallis test result was also significant when comparing the age of male cases with RAI ≥ 50 and the age of male cases with an RAI < 50 (Table 3, Fig. 2). For females, however, the test result was not significant (Table 3, Fig. 3).

**Table 2:** Overview of the Kruskal–Wallis seasonal Test. Significance at 95% confidence level (p < 0.05) is indicated with an asterisk

Caseload spring: 105	Caseload summer: 55	Caseload autumn: 41	Caseload winter: 81	
Tested groups			Result	
Summer vs. winter			H	0
			P	0.98
Spring vs. summer			H	2.56
			P	0.11
Autumn vs. spring			H	1.17
			P	0.28
Autumn vs. summer			H	0.12
			P	0.73
Autumn vs. winter			H	0.13
			P	0.72
Spring vs. winter			H	3.36
			P	0.07

**Table 3:** Overview of the statistical results for sex, RAI and age. Significance at 95% confidence level (p < 0.05) is indicated with an asterisk

Tested groups	Result	
Sex vs. RAI	H	2,33
	P	0,13
RAI of all young cases (< 50 yrs) vs. RAI of all old cases (≥ 50 yrs)	H	2.86
	P	0.09
Age of cases with RAI ≥ 50 vs. age of cases with RAI < 50	H	4.93
	P	0.03*
Age of male cases with RAI ≥ 50 vs. age of male cases with RAI < 50	H	8.21
	P	0.004*
Age of female cases with RAI ≥ 50 vs. age of female cases with RAI < 50	H	0.29
	P	0.59

The linear regression of RAI vs. age in men had a slope of 0.35, suggesting a weak positive correlation. RAI vs. age in women had a very weak correlation, with a slope of only 0.1 (see Figs. 2 and 3). Male cases aged ≥ 50 years tended to have a higher RAI, suggesting that they were found in more advanced stages of decomposition than female cases. In the female group, there was no correlation between age and the RAI / state of decomposition.

## Discussion

Our aim was to evaluate the correlation between seasonal influences, age and sex and the state of decomposition by using the RAI as a proxy for the extent of decomposition.

### Seasonal effects on the state of decomposition

Our data showed no correlation between the RAI and season in urban locations when the bodies are mainly discovered indoors. The subjective perception among forensic pathologists that there tend to be more cases of advanced decomposition in summer cannot be confirmed in our study population. This may be attributed to the site of discovery of the body, as it was indoors in the majority of cases, and indoor environments have a relatively consistent temperature of approximately 18–23 °C throughout the whole year. Even the long summer holidays, that could have had an influence on the body discoverability of distant relatives, showed no significant effect for summer season.

### Seasonal effects on body detection rates (with RAI > 0)

We found a slightly increased case number in spring, but the difference was not significant. Sociological studies found that mortality rates decreased before events people deemed important, such as birthdays, or religious, political or domestic ceremonies, and increased by the same amount afterwards [21, 22]. In terms of completed suicides, in an Italian study, a peak (males > females) was observed in spring [23–25], whereas in Switzerland, suicide peaks were observed in winter (January) and summer (June) [26]. We could not find a similar correlation in our urban study group since the case number of suicides with intact bodies (open trauma or polytrauma were exclusion criteria in our study) was too small (31 cases).

### Social implications of advanced states of decomposition

There was a significant correlation between higher RAI and older age in males. The older the male was at the time of death, the higher the RAI and thereby more advanced the state of decomposition of his body was at the time of discovery. Advanced cadaveric alterations allow for not only estimating the PMI but also interpreting the social environment of the deceased. The more time that passes until a body is found, the less likely it is that the person had a dense social network; otherwise, the body would have been discovered earlier. As previous studies have shown, adequate, meaningful social relationships are a major contributor in survival, where survival is increased by up to 50% over 7.5 years in those with strong social networks compared to individuals with insufficient or poor relationships [27]. Loneliness and social isolation increase with age [28], and they are associated with an increased mortality risk [29] and an earlier than normal death [27]. Men of advanced age rely heavily on the company of their spouses and little on their social environment for support [30, 31]. Our results might be seen as an expression of this sociological phenomenon.

### Limitations

Even though Egger et al. described a consistency between gas accumulation and RAI scores [14], and decomposition gas is known to increase with advancing decomposition, the RAI has not yet been validated for its actual correlation with the postmortem interval.

Furthermore, RAI defines the amount of intracorporeal gas at the time of PMCT, not at the time of the external examination on site. In our population, the time difference between external examination and PMCT was 0.77 days (standard deviation 0.79). Only in cases with a PMI of days rather than hours, this time difference of some hours might be neglected.

The population in this study consisted of mainly urban residents, with most deceased being found indoors, and thereby representing a different environment than for example, that of a rural area. Our study population, therefore, does not allow for a generalization regarding seasonal influences on state of decomposition. Furthermore, the evaluation was limited to autopsy cases and did not include cases that underwent only forensic examination on site (release of the body on site by the state attorney).

## Conclusion

The main purpose of a forensic postmortem investigation is of the determination of the cause and manner of death and possible third-party involvement in a suspected criminal offense. It is our opinion that beyond this major primary task, important additional aspects can be derived from the collected data, such as insights into sociopolitical backgrounds and relationships.

## Keypoints:

1. Dead bodies exhibit a variable range of changes with advancing decomposition; in our study, the radiological alteration index (RAI) in postmortem computed tomography was used as a proxy for the state of decomposition.
2. Seasonal effects had no significant influence on the RAI in our urban study population with bodies mostly being discovered indoors.
3. A significant correlation was found between age and RAI; a higher RAI for the older male sample was found when compared to the female sample, maybe reflecting that a dense social network is often missing in males.
4. Advanced cadaveric alterations allow not only for estimation of the PMI but also allow interpretation of the social environment of the deceased.
5. Autopsy reports contain valuable information about the deceased related to sociological topics.

## References

[1] Clark MA, Worrell MB, Pless JE (1997). Forensic taphonomy: the postmortem fate of human remains.

[2] Aydin B, Colak B, Balci Y, Demirüstü C (2010). Consistency of postmortem interval estimations of physicians using only postmortem changes of putrefied dead bodies. Am J Forensic Med Pathol.

[3] Sabucedo AJ, Furton KG (2003). Estimation of postmortem interval using the protein marker cardiac Troponin I. Forensic Sci Int.

[4] Henssge C, Madea B (2007). Estimation of the time since death. Forensic Sci Int.

[5] Megyesi MS, Nawrocki SP, Haskell NH (2005). Using accumulated degree-days to estimate the postmortem interval from decomposed human remains. J Forensic Sci.

[6] Moffatt C, Simmons T, Lynch-Aird J (2016). An improved equation for TBS and ADD: Establishing a reliable postmortem interval framework for casework and experimental studies. J Forensic Sci.

[7] Colombo P (2011). Estimation of the time since death in the later postmortem interval: proposition of a compound method based on a Europe-wide survey.

[8] Grabherr S, Heinemann A, Vogel H, Rutty G, Morgan B, Woźniak K (2018). Postmortem CT angiography compared with autopsy: A forensic multicenter study. Radiology.

[9] Ampanozi G, Halbheer D, Ebert LC, Thali MJ, Held U (2019). Postmortem imaging findings and cause of death determination compared with autopsy: a systematic review of diagnostic test accuracy and meta-analysis. Int J Legal Med.

[10] Thali MJ, Jackowski C, Oesterhelweg L, Ross SG, Dirnhofer R (2007). VIRTOPSY – The Swiss virtual autopsy approach. Leg Med.

[11] Aghayev E, Christe A, Sonnenschein M, Yen K, Jackowski C, Thali MJ (2008). Postmortem imaging of blunt chest trauma using CT and MRI: comparison with autopsy. J Thorac Imaging.

[12] Bolliger SA, Thali MJ, Ross S, Buck U, Naether S, Vock P. Virtual autopsy using imaging: bridging radiologic and forensic sciences. A review of the Virtopsy and similar projects. Eur Radiol. 2008;18:273–82. 10.1007/s00330-007-0737-4 17705044

[13] Christe A, Flach P, Ross S, Spendlove D, Bolliger S, Vock P (2010). Clinical radiology and postmortem imaging (Virtopsy) are not the same: Specific and unspecific postmortem signs. Leg Med (Tokyo).

[14] Egger C, Vaucher P, Doenz F, Palmiere C, Mangin P, Grabherr S (2012). Development and validation of a postmortem radiological alteration index: the RA-Index. Int J Legal Med.

[15] Adlam RE, Simmons T (2007). The effect of repeated physical disturbance on soft tissue decomposition–are taphonomic studies an accurate reflection of decomposition?. J Forensic Sci.

[16] Rodriguez W, Bass W (1983). Insect activity and its relationship to decay rates of human cadavers in east Tennessee. J Forensic Sci.

[17] Haskell NH, Hall RD, Cervenka VJ, Clark MA (1997). On the body: insects’ life stage presence, their postmortem artefacts.

[18] Udell JA, Steg PG, Scirica BM, Smith SC, Ohman EM, Eagle KA (2012). Living alone and cardiovascular risk in outpatients at risk of or with atherothrombosis. Arch Intern Med.

[19] Steptoe A, Shankar A, Demakakos P, Wardle J (2013). Social isolation, loneliness, and all-cause mortality in older men and women. Proc Natl Acad Sci USA.

[20] Flach PM, Gascho D, Schweitzer W, Ruder TD, Berger N, Ross SG (2014). Imaging in forensic radiology: an illustrated guide for postmortem computed tomography technique and protocols. Forensic Sci Med Pathol.

[21] Phillips DP, Feldman KA (1973). A dip in deaths before ceremonial occasions: some new relationships between social integration and mortality. Am Sociol Rev.

[22] Phillips DP, Smith DG (1990). Postponement of death until symbolically meaningful occasions. JAMA.

[23] Chew KS, McCleary R (1995). The spring peak in suicides: a cross-national analysis. Soc Sci Med.

[24] Maes M, Cosyns P, Meltzer HY, De Meyer F, Peeters D (1993). Seasonality in violent suicide but not in nonviolent suicide or homicide. Am J Psychiatry.

[25] Ajdacic-Gross V, Wang J, Bopp M, Eich D, Rössler W, Gutzwiller F (2003). Are seasonalities in suicide dependent on suicide methods?. A reappraisal Soc Sci Med.

[26] Ajdacic-Gross V, Lauber C, Sansossio R, Bopp M, Eich D, Gostynski M (2007). Seasonal associations between weather conditions and suicide–evidence against a classic hypothesis. Am J Epidemiol.

[27] Holt-Lunstad J, Smith TB, Layton JB (2010). Social relationships and mortality risk: a meta-analytic review. PLoS Med.

[28] Jylhä M (2004). Old age and loneliness: cross-sectional and longitudinal analyses in the Tampere Longitudinal Study on Aging. Can J Aging.

[29] Luo Y, Hawkley LC, Waite LJ, Cacioppo JT (2012). Loneliness, health, and mortality in old age: a national longitudinal study. Soc Sci Med.

[30] Antonucci TC, Akiyama H (1987). An examination of sex differences in social support among older men and women. Sex Roles.

[31] Srivastava S, McGonigal KM, Richards JM, Butler EA, Gross JJ (2006). Optimism in close relationships: How seeing things in a positive light makes them so. J Pers Soc Psychol.

